# Using Copy Number Alterations to Identify New Therapeutic Targets for Bladder Carcinoma

**DOI:** 10.3390/ijms17030271

**Published:** 2016-02-24

**Authors:** Donatella Conconi, Elena Sala, Giorgio Bovo, Guido Strada, Leda Dalprà, Marialuisa Lavitrano, Angela Bentivegna

**Affiliations:** 1 School of Medicine and Surgery, University of Milano-Bicocca, 20900 Monza, Italy; donatella.conconi@unimib.it (D.C.); leda.dalpra@unimib.it (L.D.); marialuisa.lavitrano@unimib.it (M.L.); 2 Medical Genetics Laboratory, San Gerardo Hospital, 20900 Monza, Italy; elena.sala@hsgerardo.org; 3 Department of Pathology, San Gerardo Hospital, 20900 Monza, Italy; g.bovo@hsgerardo.org; 4 Urology Division, Bassini Icp Hospital, 20092 Cinisello Balsamo, Italy; guido.strada@icp.mi.it

**Keywords:** bladder cancer, transitional cell carcinomas, therapeutic targets, cancer stem cells, copy number alterations

## Abstract

Bladder cancer represents the ninth most widespread malignancy throughout the world. It is characterized by the presence of two different clinical and prognostic subtypes: non-muscle-invasive bladder cancers (NMIBCs) and muscle-invasive bladder cancers (MIBCs). MIBCs have a poor outcome with a common progression to metastasis. Despite improvements in knowledge, treatment has not advanced significantly in recent years, with the absence of new therapeutic targets. Because of the limitations of current therapeutic options, the greater challenge will be to identify biomarkers for clinical application. For this reason, we compared our array comparative genomic hybridization (array-CGH) results with those reported in literature for invasive bladder tumors and, in particular, we focused on the evaluation of copy number alterations (CNAs) present in biopsies and retained in the corresponding cancer stem cell (CSC) subpopulations that should be the main target of therapy. According to our data, *CCNE1*, *MYC*, *MDM2* and *PPARG* genes could be interesting therapeutic targets for bladder CSC subpopulations. Surprisingly, *HER2* copy number gains are not retained in bladder CSCs, making the gene-targeted therapy less interesting than the others. These results provide precious advice for further study on bladder therapy; however, the clinical importance of these results should be explored.

## 1. Introduction

Bladder cancer represents the ninth most widespread malignancy with 429,000 new cases and about 165,000 fatalities in 2012 (2% of the total number of cancer deaths). It occurs in men more than in women, with a sex ratio of 3.5 [1]. More than 90% of these tumors are transitional cell carcinomas (TCC, also urothelial carcinoma). This tumor is characterized by the presence of two different clinical and prognostic subtypes: non-muscle-invasive bladder cancers (NMIBCs) and muscle-invasive bladder cancers (MIBCs). At diagnosis the majority are non-muscle-invasive, papillary and low grade. NMIBC has the highest recurrence rate (50%–80%) of any carcinoma and, consequently, this is the most expensive carcinoma per patient between diagnosis and death [2] because of repetitive and costly follow-up. However, the prognosis is usually good, with only 10%–15% progressing towards invasion while the five-year survival rate is 90%. On the contrary, about 20% of cancers present muscle invasion at diagnosis, an unfavorable outcome with a survival rate after five years below 50% [3] and common progression to metastasis.

For this reason, two different potential pathways have been proposed. The onset of NMIBCs may be derived via simple hyperplasia and minimal dysplasia through the loss of heterozygosity of chromosome 9 and activating mutations of *FGFR3*, *PIK3CA* and *STAG2*. Invasive carcinomas could be due to *TP53* mutation in addition to chromosome 9 deletions, but generally without *FGFR3* mutations via flat dysplasia and carcinoma *in situ* [4].

A recent study has combined statistical analysis and computational modeling to identify co-occurrence and mutual exclusivity of genes involved in bladder cancer [5]. Authors confirmed that *FGFR3* and *PIK3CA* mutations along with *CDKN2A* deletions are more associated with the noninvasive pathway, whereas *EGFR*, *E2F3* amplifications and *TP53* mutations characterized the invasive pathway.

Despite improvements in knowledge, treatment has not advanced significantly in recent years, with the absence of new therapeutic targets. NMIBC treatment options include: removing the tumor(s) via transurethral resection with fulguration, eventually followed by instillation of intravesical chemotherapy and possibly periodic intravesical instillations of bacillus Calmette–Guérin for high risk of recurrent tumors [6]. Standard treatment for patients with MIBCs is either neoadjuvant multiagent cisplatin–based chemotherapy and then radical cystectomy and urinary deviation, or radiation therapy associated with chemotherapy [6]. Because of the limitations of current therapeutic options, the greater challenge will be to identify biomarkers for clinical application.

We have been studying bladder cancer genetics for many years. We firstly reported the isolation and biological characterization of putative bladder cancer stem cell (CSC) populations from primary TCCs [7]. CSCs expand as clonally derived spheres (urospheres) with extensive proliferation and self-renewal capabilities. These cells showed positivity for stem cell markers and they could differentiate in the presence of serum. Cytogenetic data indicated an enrichment of hypo- or near-diploid cells, without the complexity of fresh tumors. Subsequently, we drew a comparison between the results of the UroVysion test executed on freshly isolated nuclei and on formalin-fixed paraffin-embedded tissues from 22 TCCs and we found no significant differences. Then, from the comparison between array comparative genomic hybridization (array-CGH) findings and the specific chromosomal data of the UroVysion test, we proved that it is still recommended to apply these two synergistic techniques, as the former is able to detect genome-wide alterations, but the second can preserve the characteristics of the individual cells [8].

However, our most interesting study concerned the comparison between array-CGH profiles of CSCs and the primary biopsy, to evaluate if differences in the genomic signature already exist in the initial steps of low grade and high grade tumors [9]. We found that CSCs isolated from low grade biopsies are highly rearranged compared to the primary biopsy, with an incommensurate number of genomic losses. This seems to be an essential characteristic which diversifies the two types of tumor, not the result of alterations occurring by chance in culture. Our approach allowed us to show that the genomic profile of low grade tumors differs from high grade tumors also in the initial steps of tumorigenesis; furthermore, a subset of low grade tumors showed a major disposition to mislay genomic regions.

These results provide precious information on bladder carcinogenesis and may be useful for the identification of personalized therapy and of potential targetable biomarkers.

## 2. Results and Discussion

In this work, we compared our array-CGH results with those reported in literature for invasive bladder tumors (Table 1); in particular, we focused on the comparison of selected genes identified by the Cancer Genome Atlas analysis [10,11] to be the most significant in this type of tumor. Our approach could lead to detecting potential therapeutic targets through the evaluation of copy number alterations (CNAs) both in biopsies and in the corresponding isolated CSC subpopulations. For example, *CCNE1* (cyclin E1) gene amplification has been reported in 9%–12% (Table 1) of bladder cancer, but in our cases a higher percentage of copy number gain in biopsies (25%) and especially in CSC subpopulations (60%) occurred. The presence of gain in more than half of CSC samples made us speculate that it might be considered an interesting therapeutic target. Currently, amplification of *CCNE1* is considered a well-defined target in high grade serous ovarian cancer [12]; however, in bladder cancer it was only identified as a potential prognostic marker [13].

*MYC* oncogene amplification has been reported in bladder cancers (13%, Table 1): our results showed the same percentage of mosaic gain in invasive biopsies (12.5%) whereas a higher percentage of mosaic gain (40%) was found in the corresponding CSC subpopulations. Recently, the effect of intravesical instillation of *MYC* inhibitor on orthotropic bladder tumor growth has been reported [14], confirming its role as a promising target for bladder cancer therapy, even if there are no current clinical trials (http://clinicaltrials.gov; https://www.clinicaltrialsregister.eu).

Also, *MDM2*, a negative regulator of tumor suppressor p53, has been reported as a potential therapeutic target in urothelial carcinoma [15] and its amplification was found in 9% of invasive tumors (Table 1). Interestingly, our biopsies showed no amplifications, but CSC subpopulations had a 40% copy number gain, proposing a possible use of *MDM2* inhibitors, which are in current clinical trials, in bladder cancer therapy [16].

Human epidermal growth factor receptor 2 (*HER2*) overexpression is a target of anti-*HER2* therapies for amplified breast cancer. Our array-CGH results showed a higher percentage of copy number gain both in low grade non-infiltrating (LGNI) and high grade infiltrating (HGIN) tumors with respect to published data (30% and 37.5%, respectively, *versus* 7% and 5%, Table 1). Recent works have evaluated the role of *HER2* in bladder cancer and also several trials are currently investigating the possible benefit of targeted therapies for patients [17].

**Figure 1:**
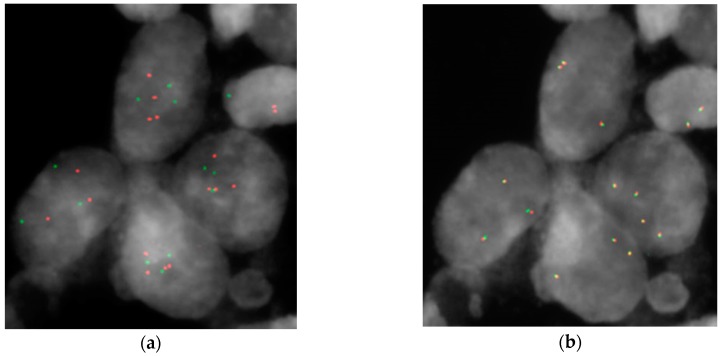
Examples of fluorescence in situ hybridization (FISH) analysis on formalin-fixed, paraffin-embedded (FFPE) tissues on the same tumoral area (case 27): (**a**) *Her*-2/*Neu* (17q12)/SE17 (SE: satellite enumeration) probe (human epidermal growth factor receptor 2 (*HER2*) red, centromere 17green); (**b**) PPARγ Break probe. Yellow dots represent the fusion of green and red signals of the dual color split probe.

For this reason, we decided to substantiate these results on another set of tumors with the fluorescence *in situ* hybridization (FISH) technique (Figure 1). We considered samples positive for *HER2* amplification that showed more than 10% of cells with an *HER2*/CEP17 (centromere 17) ratio higher than two or with more than six *HER2* signals per nucleus independent of CEP17 signals [18]. We found that 66.7% of tumors (both LGNI and HGIN) were positive for *HER2* amplification (Table 2). This disparity with array-CGH data could be explained by the peculiarity of the two techniques and the different information provided by them, as already reported in our previous paper [8].

Then our cohort of Italian patients showed a significant variance in *HER2* amplification with respect to published data. In particular, The Cancer Genome Atlas Project identified 7% amplification in 131 patients with high grade urothelial cancer [10] and other studies displayed a frequency of amplification ranging from 5% to 14%, with the exception of 42% detected in the infrequent micropapillary histological variant [4]. However, a recent study on two different cohorts of patients proved a significant difference in frequencies of *HER2* amplification between the Spanish and Greek patients, with 20% and 4%, respectively. These results suggest that *HER2* amplification can change between populations and promote the hypothesis that etiologic heterogeneity can lead to these differences [19].

To evaluate if *HER2* may be a therapeutic target in a subgroup of urothelial carcinomas, we analyzed the results obtained in the CSCs subpopulation. Comparison between biopsies and the corresponding CSCs showed that *HER2* copy number gains are not retained in CSCs except for one case (Table 3). This result could suggest the hypothesis that *HER2* amplification is not present in bladder cancer tumor-initiating cells. Moreover, the majority of *HER2* amplifications detected in biopsies are as a mosaic (Table 3), so a low level of mosaicism amplification could be also in CSCs, but not detectable by the array-CGH technique.

The most known gene involved in bladder cancer is *CDKN2A*. Our array-CGH results are in agreement with the literature, with a 40% loss in LGNI biopsies (30% complete loss) and 50% in HGIN biopsies (12.5% complete loss); instead, FISH analysis performed on another set of tumor biopsies revealed a 100% loss in LGNI (5/5 cases) and 62.5% in HGIN samples (5/8 cases) with a median number of signals less than two (Table 2). *CDKN2A* loss was also maintained in CSC subpopulations (30% and 60% in LGNI and HGIN, respectively), denoting its important role in cancer onset and progression (Table 3).

Finally, *PPARG* results were analyzed. We reported *PPARG* amplification in three samples derived from the same patient with multifocal non-muscle-invasive bladder cancer, giving a novel proof on the *PPARG* role in onset and support of bladder cancer, and also a potential explanation for the monoclonal origin of multifocality [20].

*PPARG* amplification has been reported in 14%–17% of bladder cancer; our HGIN biopsies showed 12.5% amplification but also 50% copy number gain (Table 1). FISH analysis performed on another set of tumor biopsies revealed a 20% of gain in LGNI (1/5) and 100% in HGIN samples (8/8) with a median number of signals greater than two (Table 2). *PPARG* gain was maintained in 40% of CSCs, only as mosaic gain (Table 3).

## 3. Materials and Methods

### 3.1. Tumor Specimens

TCC specimens were collected from 33 patients that underwent transurethral resection at a single center, as previously reported [9]. Staging and grading were done according to the World Health Organization Consensus Classification by a pathologist. Samples were classified as high or low grade (HG or LG) and muscle infiltrating or non-muscle-infiltrating (IN or NI) (see Table 2 and Table 3). No patient has been treated before surgery.

This study was approved and founded by Direzione Generale Sanità Regione Lombardia and presented by General Director and ethic commitment of ICP Hospital Bassini (Cinisello Balsamo, Italy), as previously reported [9]. Written informed consent was obtained before tissue collection.

### 3.2. Array Comparative Genomic Hybridization (Array-CGH)

Genomic DNA extraction, sample preparation, slide hybridization and analysis were performed using SurePrint G3 Human CGH Microarray 8x60K (Agilent Technologies, Santa Clara, CA, USA) following the manufacturer’s recommendations. The arrays were scanned at 2-μm resolution and analyzed using Feature Extraction v10.7 and Agilent Genomic Workbench v5.0 software (Agilent Technologies), as previously reported [9].

### 3.3. Fluorescence in Situ Hybridization (FISH)

FISH analysis on formalin-fixed, paraffin-embedded (FFPE) tissue sections was performed using *Her-2/Neu* (17q12)/SE17 probe (Kreatech Diagnostics, Amsterdam, The Netherlands), *PPARγ* (3p25) Break probe (Kreatech Diagnostics) and UroVysion bladder cancer kit (Vysis, Wiesbaden, Germany) according to the manufacturer’s instructions. HER2 amplification is considered for samples that show more than 10% of cells with *HER2*/CEP17 ratio greater than two or with more than six *HER2* signals per nucleus (ASCO international guidelines for breast cancer therapy) [18]. All digital images were captured using a Leitz microscope (Leica DM 5000B, Leica Microsystems GmbH, Wetzlar, Germany) equipped with a charge coupled device (CCD) camera (Leica Microsystems) and analyzed by means of Chromowin software (Tesi Imaging, Milano, Italy).

## 4. Conclusions

In conclusion, our approach allowed us to evaluate genes with copy number alterations in biopsies that are retained in the corresponding CSC subpopulations, which should be the main target of therapy. According to our data, *CCNE1*, *MYC*, *MDM2* and *PPARG* genes could be interesting therapeutic targets for bladder CSC subpopulations in order to overcome the limitations of current therapeutic options. Surprisingly, *HER2* copy number gains are not retained in bladder CSCs, making the gene-targeted therapy less interesting than the others. These results provide precious advice to further study on bladder cancer therapy; however, the clinical importance of these results should be explored.

## Figures and Tables

**Table 1:** Array comparative genomic hybridization (array-CGH) results. Comparison with literature.

Genes	Nature [10]		Clin Cancer Res [11]		Biopsies		CSC Subpopulation		Biopsies		CSC Subpopulation	
	% Loss HGIN	% Gain HGIN	% Loss IN	% Gain IN	% Loss LGNI	% Loss HGIN	% Loss LGNI	% Loss HGIN	% Gain LGNI	% Gain HGIN	% Gain LGNI	% Gain HGIN
*CCND1* *	-	10 A°	-	11 A	-	-	10 M	20 M	20 A 30 M	12.5 A 12.5 NM 12.5 M	40 A 20 M	20 A 20 NM
*E2F3/SOX4*	-	20 A°	-	18 A	-	-	-	-	-	12.5 NM	-	20 NM
*EGFR*	-	11 A°	-	7 A	-	-	10 M	-	20 NM 10 M	12.5 M	30 NM	20 M
*PPARG*	-	17 A°	-	14 A	-	-	-	-	-	12.5 A 25 NM 25 M	-	40 M
*PVRL4* *	-	19 A°	-	17 A	-	-	10 CL 20 NM	20 NM	10 NM 10 M	75 M	30 NM 10 M	40 M
*YWHAZ* *	-	22 A°	-	22 A	-	-	-	-	10 NM	25 NM	10 NM	40 NM
*MDM2*	-	9 A°	-	9 A	-	-	10 M	-	20 M	-	10 M	20 NM 20 M
*HER2*	-	7 A°	-	5 A	-	-	20 M	-	20 M 10 NM	37.5 M	10 M	-
*YAP1*	-	4 A°	-	ni	10 M	12.5 M	-	-	-	12.5 M	-	25 M
*CCNE1*	-	12 A°	-	9 A	-	12.5 M	-	-	-	25 M	-	60 M
*MYC*	-	13 A°	-	13 A	-	12.5 M	10 NM 10 M	-	-	12.5 M	10 M	40 M
*FGFR3* *	-	3 A°	-	4 A	-	12.5 M	20 M	20 M	10 A 40 M	12.5 M	10 A 10 M	20 M
*MYCL1* *	-	6 A°		6 A	-	-	-	-	10 M	12.5 M	10 NM	-
*BCL2L1*	-	11 A°	-	10 A	-	-	10 CL 20 NM	-	50 M	12.5 A 12.5 NM 37.5 M	10 A 20 NM 10 M	20 NM
*BEND3* *	-	ni	-	3 A	-	37.5 NM	-	-	10 M	25 NM	20 NM 10 M	-
*BIRC3*	-	ni	-	4 A	20 M	37.5 M	10 NM 10 M	20 M	-	-	10 M	20 NM 20 M
*GDI2* *	-	ni	-	9 A	30 M	12.5 M	10 M		10 NM 10 M	25 M	10 NM	60 M
*PRKCI*	-	ni		4 A	-	-	-	-	-	12.5 M	-	-
*SOX4*	-	ni		18 A	-	-	-	-	-	12.5 NM	-	20 NM
*CDKN2A*	47 D°	-	43 D		30 CL 10 M	12.5 CL 12.5 NM 25 M	30 CL	20 CL 40 M	-	-	-	-
*PTEN*	13 D°	-	13 D	-	-	-	-	-	10 M	-	-	20 NM
*NCOR1*	25 D°	-	24 D	-	-	25 M	-	20 M	-	-	-	-
*CREBBP*	13 D°	-	16 D	-	20 M	25 M	10 M	-	-	37.5 M	-	-
*RB1*	14 D°	-	17 D	-	-	12.5 M	40 M	20 M	-	12.5 M	-	-
*ARID1A*	ni	-	5 D	-	10 M	-	-	-	-	-	-	-
*FHIT*	ni	-	13 D	-	10 M	12.5 M	10 M	20 M	-	12.5 M	-	-
*IKZF2*	ni	-	15 D	-	-	50 M	-	20 M	10 M	-	-	-
*LRP1B*	ni	-	17 D	-	-	37.5 M	-	20 M	-	-	-	-
*PDE4D*	ni	-	22 D	-	-	25 M	-	20 M	-	-	-	-
*WWOX*	ni	-	15 D	-	-	12.5 M	-	-	-	-	-	-

A°: copy number > 3; D°: copy number < 1.5; A: amplification; CL: complete loss; M: mosaic; NM: non-mosaic; D: deletion; ni: no information; LGNI: low grade non-infiltrating; HGIN: high grade infiltrating; CSC: cancer stem cell; *: only one or two probes.

**Table 2:** Fluorescence in situ hybridization (FISH) on formalin-fixed, paraffin-embedded (FFPE) tissues.

**HGIN**	***HER2* % of Amplified Cells**	***PPARG* > Two Signals**	***CDKN2A* < Two Signals**
19	22%	32% m = 2.36	100% m = 0.08
20	6%	-	85% m = 1.02
21	84%	56.4% m = 3	58% m = 1.19
22	80%	50% m = 2.8	6% m = 2.94
23	30%	63.6% m = 2.97	10% m = 2.27
24	2.5%	48% m = 2.66	-
25	3.3%	42% m = 2.4	100% m = 0.02
26	20%	28% m = 2.34	7% m = 2.72
27	37.5%	87.5% m = 3.7	43% m = 1.83
**LGNI**	***HER2* % of Amplified Cells**	***PPARG* > Two Signals**	***CDKN2A* < Two Signals**
28	8%	4% m = 2.02	100% m = 0.17
29	10%	-	61% m = 1.28
30	13%	10% m = 1.6	100% m = 0
31	32%	2% m = 1.72	58% m = 1.21
32	14%	38% m = 2.52	99% m = 0.03
33	30%	4% m = 1.76	-

m: mean number of signals in all analyzed nuclei; LGNI: low grade non-infiltrating; HGIN: high grade infiltrating.

**Table 3:** Array-CGH results. Comparison between biopsies and cancer stem cells.

Histotype	CASE n°	Biopsies			Cancer Stem Cells		
		*HER2*	*PPARγ*	*CDKN2A*	*HER2*	*PPARγ*	*CDKN2A*
**LGNI**	1	disomy	disomy	disomy	disomy	disomy	disomy
	2	disomy	disomy	complete loss	disomy	disomy	complete loss
	3	disomy	disomy	disomy	mosaic loss	disomy	disomy
	4	non mosaic gain	disomy	disomy	mosaic loss	disomy	disomy
	5	mosaic gain	disomy	mosaic loss	mosaic gain	disomy	disomy
	6	mosaic gain	disomy	disomy	disomy	disomy	disomy
	7	disomy	disomy	disomy	disomy	disomy	disomy
	8	disomy	disomy	complete loss	disomy	disomy	complete loss
	9	disomy	disomy	disomy	disomy	disomy	disomy
	10	disomy	disomy	complete loss	disomy	disomy	complete loss
**HGIN**	11	disomy	non mosaic gain	mosaic loss	disomy	disomy	disomy
	12	mosaic gain	mosaic gain	complete loss	disomy	mosaic gain	mosaic loss
	13	disomy	disomy	mosaic loss	disomy	disomy	mosaic loss
	14	mosaic gain	disomy	non mosaic loss	disomy	disomy	complete loss
	15	disomy	non mosaic gain	disomy	disomy	mosaic gain	disomy
	16	disomy	amplification	disomy	-	-	-
	17	disomy	disomy	disomy	-	-	-
	18	mosaic gain	disomy	disomy	-	-	-

n**°**: number.

## Abbreviations

TCC: transitional cell carcinomas
NMIBC: non-muscle-invasive bladder cancer
MIBC: muscle-invasive bladder cancer
CSC: cancer stem cell
CGH: comparative genomic hybridization
CNA: copy number alteration
LGNI: low grade non-infiltrating
HGIN: high grade infiltrating
FISH: fluorescence *in situ* hybridization
